# Supplementary material for: Braille letter reading: A benchmark for spatio-temporal pattern recognition on neuromorphic hardware
Source: Front Neurosci. 2022 Nov 11;16:951164. doi: 10.3389/fnins.2022.951164 (PMC9695069; doi:10.3389/fnins.2022.951164)
Supplement: Supplementary file 1 [file Data_Sheet_1.PDF]

Supplementary Material

1 SUPPLEMENTARY DATA

1.1 Time-Series Classification

More details regarding the architectures used for time-series classification:

FCN: Mainly a CNN but without local pooling layers, which keeps the length of the time series unchanged. The last FC layer is replaced by a GAP layer, helping to identify which of the time series contributed the most to the classification and reducing the number of parameters. The network contains 3 convolutional blocks with a stride of 1, each performing three operations: 1) convolution, 2) batch normalization, and 3) Rectified Linear Unit (ReLU) activation function. The result of the third convolutional block is averaged over the whole time dimension. The first convolution contains 128 filters with a filter length of 8, the second contains 256 filters with a filter length of 5, and the last contains 128 filters with a filter length of 3.

ResNet: The network is composed of 11 layers where the first 9 are convolutional layers, of which every 3 layers are forming a residual block. Short cuts between these residual blocks are the main characteristic and difference to FCNs. These blocks are followed by a GAP layer, averaging the time series across the time dimension, and a final softmax layer, containing neurons equal to the number of classes in the dataset. The convolutions in the residual blocks have a filter length fixed to 64 with batch normalization followed by ReLU as an activation function. The filter length in the residual blocks is 8, 5, and 3 respectively.

Encoder: A hybrid deep CNN inspired by FCNs, whereas the GAP layer is replaced by an attention layer. The first three layers are convolutional layers. The first has 128 filters with a length of 5, the second 256 filters with a length of 11, and the third 512 filters with a length of 21. Each convolution is followed by an instance normalization operation and fed to a PReLU. The output of the PReLU is followed by a dropout operation with a rate of 0.2 and a final max-pooling of length 2. Finally, after the attention layer, a softmax classifier with the number of neurons equal to the classes in the dataset is used.

TCNN: The TCNN differs in 3 main points from the previously described networks. First, instead of using the cross-entropy loss function, MSE is used, leading to a FC final layer with sigmoid as activation function. Second, local average pooling instead of local max pooling is used and one convolution is applied to each dimension of the MTS. Third, the final classifier is FC directly to the second convolution. Two convolutional layers with filter lengths 6 and 12 are followed by a local average pooling operation with a length of 3 and sigmoid as an activation function. The convolutions are applied to all dimensions. The final FC layer has neurons equal to the number of classes in the dataset and sigmoid as an activation function. Further, the output of the second convolution is directly fed as input to the FC layer.

Inception: The network is composed of two residual blocks, each containing three inception modules. Each inception module contains three main parts. The first part is a bottleneck layer, which performs convolution to represent the input according to the user-defined size, the default value is 32, to reduce the input dimensionality. The second part is three parallel sliding filters with an increasing length of 10, 20, and 40, which are applied to the output of the previous bottleneck layer. The third part is a max pooling layer, performed on the input of the inception module, which is followed by a bottleneck layer. The output of the three filters together with this bottleneck layer are concatenated and form the output of a module.

The two residual blocks are followed by a GAP layer and a final FC layer with neurons equal to the number of classes in the dataset.

1.2 Computing parameters for the LSTM

The calculation of the number of parameters in the RSNN was made according to

$$\text{snn parameters} = nb\_channel \cdot nb\_input\_copies \cdot nb\_hidden + nb\_hidden^2 + nb\_hidden \cdot nb\_output \quad \text{(S1)}$$

with $nb\_channel = 24$, $nb\_input\_copies = 2$, $nb\_hidden = 450$, and $nb\_output = 27$.

The LSTM recurrent neural network is comprised of four basic gates that take as input the concatenation of the input feature vector $x_{(t-n)}$ and the previous hidden state, and can be conceptually summarized as

1. *Input gate*: it decides which new values are going to be incorporated into the cell.
2. *Forget gate*: it takes the input x_t, and the previous hidden state h_{t-1} and delivers a number between 0 and 1 for each cell state C_{t-1} indicating how much past information should be remembered.
3. *Estimated cell state*: New candidate vector created also from the input x_t and previously hidden state h_{t-1}, that is combined with the information coming from the input gate, to produce the new cell state C_t.
4. *Output gate*: it filters the new cell state and combines it with a filtered version of the input and last hidden state h_{t-1} to produce the new hidden state h_t.

Each gate has the same number of trainable parameters in the form of weight W and bias b, and can be calculated by hand as

$$\text{parameters per gate} = \underbrace{h_{size} \cdot (h_{size} + x_{size})}_{\text{number of } W_i} + \underbrace{h_{size}}_{\text{number of } b_i} \quad \text{(S2)}$$

where $(h_{size} + x_{size})$ is the resulting dimension of the concatenation between the input feature x_t and previous hidden state h_{t-1}, resulting in a total parameter count of

$$\text{LSTM parameters} = 4(h_{size} \cdot nb\_channel + h_{size} + x_{size}) + h_{size} \cdot nb\_output \quad \text{(S3)}$$

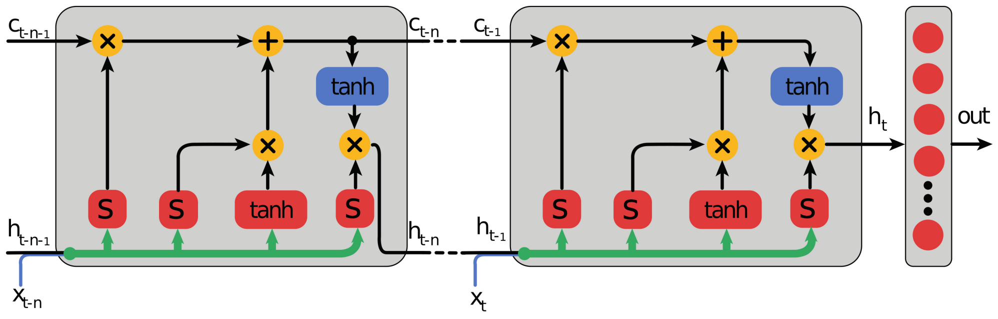

Figure S1. Implemented single layer LSTM with a fully connected of size $h \times 27$. The green line represents the concatenation operation, the red blocks represent weighted operations followed by the activation indicated in each label, the yellow is element-wise operations, and the blue is $tanh$ activation functions.

1.3 Hyperparameters optimization results

Using the best values found in the grid search for the $time\_bin\_size$ and the $nb\_input\_coopies$ parameters, and the remaining ones from the first step of the HPOs summarized in Fig. S2, we performed training and test of the resulting network five times to estimate the mean and standard deviation of test accuracy. The accuracy increases for threshold $\vartheta = 2$ and starts to decrease again for higher thresholds for the FFSNN, with a similar accuracy for the RSNN for threshold $\vartheta = 2$ and $\vartheta = 3$, but a higher variance for threshold $\vartheta = 3$. For the RSNN the smallest variance is reported for threshold $\vartheta = 2$ and the highest for threshold $\vartheta = 5$, whereas for the FFSNN the smallest variance is found for threshold $\vartheta = 5$ and the highest for threshold $\vartheta = 10$. All values are reported in Tab. S1. Further analysis of events propagated in the network during inference is then reported in Tab. S2. Here we compare for each of the layers the number of events at the inference of the whole dataset at different ϑ threshold values, the results are compared with the dataset at threshold $\vartheta = 1$. In both, the recurrent and the FFSNNs, the activity in the input layer decreases as the encoding threshold increases, while the opposite occurs for both the subsequent layers. Looking at the total number of spikes, the largest increase is observed with $\vartheta = 5$, while a threshold $\vartheta = 10$ results in the smallest increase of the network activity. Comparing the RSNN with the FFSNN, this latter is more sensitive to the threshold value: focusing on the total number of spikes, the case of $\vartheta = 2$ leads, compared to the reference case with $\vartheta = 1$, to an activity equal to $1.26\times$ for the recurrent network and equal to $2.62\times$ for the feedforward one.

Table S1. Mean and standard deviation of the RSNN and FFSNN of 5 runs per threshold for different train-test splits with the best parameters found in the two-step HPO.

	**Accuracy** (%)	
**Threshold** (ϑ)	**Recurrent**	**Feedforward**
**1**	79.9 ± 1.4	71.5 ± 0.7
**2**	80.9 ± 0.3	71.9 ± 1.2
**5**	80.9 ± 1.9	61.9 ± 0.5
**10**	72.7 ± 1.3	45.1 ± 1.4

1.4 Energy consumption approximation from SNN simulation

The total number of spikes normalized per letter in the dataset is shown in Tab. S2. The spikes have been calculated in simulation with the parameters and weights pre-trained according to Tab. 3. Similarly to the

Table S2. Number of spikes calculated as single inference of the whole dataset in each of the layers of the network trained with parameters obtained from the hyperparameter optimization. Percentages as the ratio of spikes with the equivalent in threshold 1 as reference.

	**Threshold** (ϑ)			
**Recurrent**	**1**	**2**	**5**	**10**
Input	698	426 (61%)	126 (18%)	41 (6%)
Hidden	1230	2165 (176%)	2775 (225%)	2591 (210%)
Output	371	301 (81%)	508 (137%)	257 (69%)
Total	***2298***	***2892 (126%)***	***3409 (148%)***	***2889 (126%)***
**Feedforward**	**1**	**2**	**5**	**10**
Input	698	426 (61%)	126 (18%)	41 (6%)
Hidden	1029	4881 (474%)	4917 (478%)	2817 (274%)
Output	496	507 (102%)	833 (168%)	235 (47%)
Total	***2223***	***5815 (262%)***	***5876 (264%)***	***3092 (139%)***

profile of energy consumption, as seen in the hardware implementation results, an increase in the encoding threshold, initially decreasing the number of spikes in the input layer, does not correspond with a decrease in the number of spikes in the total network.

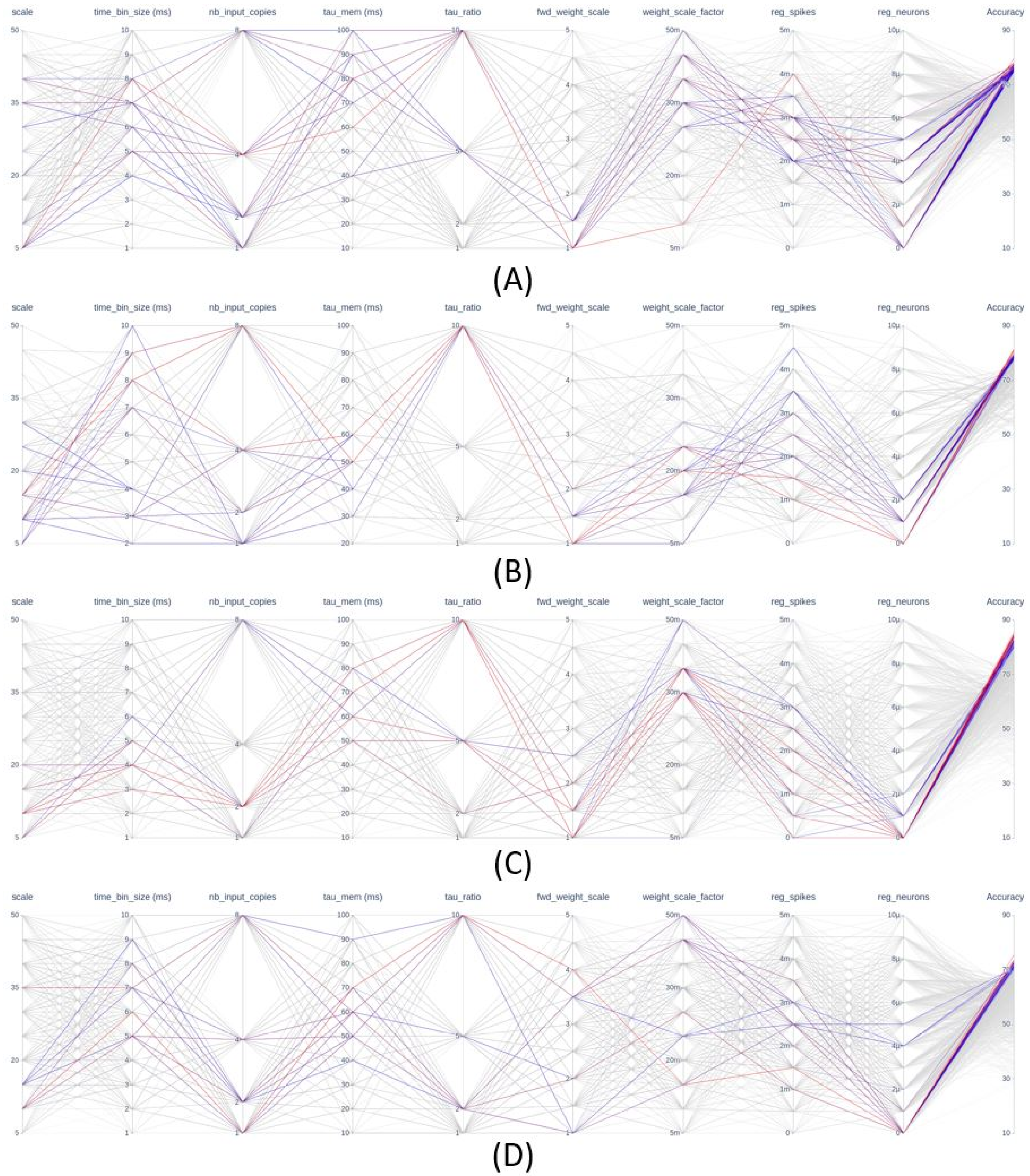

Figure S2. Summary of the hyperparameter values explored during the first step of the HPO procedure for (a) ϑ=1, (b) ϑ=2, (c) ϑ=5, and (d) ϑ=10. Highlighted are the top 10% in accuracy.
